# Intravenous sedation for adults with profound acquired brain injury undergoing dental treatment - a seven-year service evaluation

**DOI:** 10.1038/s41405-024-00289-2

**Published:** 2024-12-30

**Authors:** Mili Doshi, Ahmed Kahatab, Louise Gallagher, Ria Prasad, Richard Fitzgerald

**Affiliations:** 1 Consultant in Special Care Dentistry, Royal Hospital for Neuro-disability, London, UK; 2 Specialist Registrar in Special Care Dentistry, Surrey and Sussex NHS Health Care Trust, Redhill, UK; 3 Dental Nurse Manager, Royal Hospital for Neuro-disability, London, UK; 4 Consultant in Special Care Dentistry, Surrey and Sussex NHS Health Care Trust, Redhill, UK

**Keywords:** Special care dentistry, Dental sedation

## Abstract

**Background:**

Dental treatment may not be possible for patients with a profound acquired brain injury without pharmacological support. Intravenous (IV) sedation with midazolam is a widely accepted, safe, and effective mode of treatment for people with a disability, but there is limited evidence in this patient cohort.

**Aims:**

This evaluation aimed to review the IV sedation service for patients with profound acquired brain injury within the dental department at the Royal Hospital for Neuro-disability.

**Method:**

This study was a retrospective service evaluation of IV sedation for dental treatment over seven years. Descriptive statistics are presented.

**Results:**

In total, 303 episodes of sedation were undertaken at the Royal Hospital of Neurodisability over seven years. Ninety-two percent were graded with an American Society of Anaesthesiologists (ASA) grade 3, and one-third had a history of stroke. 91% had treatment completed successfully. Complications occurred in 7.9% of cases, but all were minor, with no resulting patient harm (e.g. a transient drop in oxygen saturation).

**Conclusion:**

Dentist-led IV sedation with midazolam is safe and effective for dental treatment for patients with a profound acquired brain injury in a specialised setting with experienced clinicians.

## Introduction

Intravenous (IV) sedation with midazolam is a widely accepted, safe, and effective technique for delivering dental care [1]. There is research available on the use of IV sedation with midazolam in ASA 1 and 2 individuals and a moderate amount of studies for those with intellectual disabilities [1–3]. There is, however, limited evidence regarding IV sedation for medically complex patients, including those who are ASA grade 3 or have a profound acquired brain injury [4, 5].

An acquired brain injury (ABI) is an injury to the brain acquired after birth and can be divided into two main categories – traumatic and non-traumatic injuries. Traumatic brain injury is an external traumatic event in which damage to the brain is sustained. Common causes of traumatic brain injury include road traffic accidents, falls, sports-related injuries, violence resulting from concussions and skull fractures, or skull penetrating injuries. Non-traumatic brain injury occurs due to internal damage to the brain, often as a result of a stroke, cancer, infection or inflammation, leading to anoxia of the brain [6]. Recovery of patients after sustaining an ABI will depend on their age, pre-injury health and degree of damage to the brain tissues. Profound ABI can lead to severe lifelong impairments impacting daily activities, memory problems, communication difficulties, multimorbidity, seizures, depression, and behaviours that challenge [6]. Some patients will have a significant disorder of consciousness (DOC). Disorders of consciousness is a state of prolonged altered consciousness, categorised into coma, vegetative, or minimally conscious state based on neurobehavioral function [7]. These patients will appear awake or asleep but have minimal or no level of consciousness and are, therefore, often unable to cooperate with mouth opening during dental examination or treatment.

One or a combination of these impairments can lead to challenges in undertaking comprehensive dental assessments, capacity assessment and safe delivery of dental treatments. Mobility issues, either due to paralysis or involuntary movements, lead to reliance on wheelchairs and mobility aids, increasing barriers to accessing dental care in general dental practice [8]. People with profound ABI often have a chin-to-chest posture, making access to the oral cavity difficult. Oral hypersensitivity, where patients resist mouth opening when touched around the face, and powerful bite reflexes often make it impossible to examine inside the mouth [9, 10]. Cognitive impairments that fluctuate may increase barriers to undertaking pain histories, assessing capacity and obtaining consent [11]. Augmentative and alternative communication aids such as whiteboards, eye-gaze-based technologies, gestures, and symbols can support communication with those with limited verbal communication or severe aphasia [12].

The Royal Hospital of Neurodisability (RHN) is a specialised care facility in southwest London, home to 250 patients with profound ABI. Specialised wards are available for patients with behavioural changes, Huntington’s disease and those on ventilators. Around 75% of residents are fully or partially fed via an enteral route due to an inability to swallow safely. 30% have a tracheostomy tube in situ, which can be temporary or long-term when patients have poor airway reflexes and pharyngeal tone, leading to a higher risk of aspiration. 98% of people use wheelchairs, often customised to provide specific head support, and most can recline [13].

An onsite dental service is commissioned to provide National Health Service (NHS) dental care to all residents and is situated within the hospital’s grounds. The clinic is staffed by three part-time consultants in special care dentistry and a dental nurse manager. Rather than hoisting patients into a dental chair, a wheelchair recliner is used to treat patients in their wheelchairs.

For this patient cohort, dental management under local anaesthetic alone may not be possible due to limited mouth opening, behaviours that challenge the provision of care (henceforth referred to as ‘challenging behaviour’), anxiety, and uncontrolled movements [14]. Therefore, undertaking dental care under general anaesthetic (GA) or conscious sedation can be beneficial [15]. GA will allow completion of dental treatment in one treatment episode but comes with added risks of morbidity/mortality, requires transfer to an acute hospital, and often inpatient admissions for this patient group due to their medical complexities [16]. Transferring patients who require frequent medication and those with tracheostomies and enteral nutrition takes significant planning and requires specialised nurses to accompany them to external appointments. It is also more stressful for patients with an ABI to recover in a hospital setting with unfamiliar staff. Waiting lists for dental general anaesthesia have increased since the COVID-19 pandemic, with competing demands for theatre access from other surgical specialities, which could result in delays in care for this cohort.

Providing dental treatment under conscious sedation, in the form of IV sedation with midazolam onsite, offers many benefits. The dentist can administer the midazolam and undertake the dental treatment working with a sedation-trained dental nurse [17]. Appointment waiting times are shorter and can be planned around the patient’s schedule, which may include physiotherapy, occupational therapy, psychology, speech and language therapy, personal care, etc. Patients often have complex medical issues, so treatment can be planned to coincide with times when they are medically optimised and postponed if they become unwell.

Midazolam, a short-acting benzodiazepine, is most widely used for its anxiolytic, sedative, muscle relaxant, amnesic, and anticonvulsant properties [18]. During sedation with midazolam, cardio-respiratory patient monitoring is carried out using pulse oximetry and the patient is monitored clinically to assess their level of sedation. To determine the depth of sedation, the clinician subjectively assesses the patient’s speech (if verbal), responsiveness, cognition, change in facial expression, muscle tone and acceptance of treatment. The main disadvantage of midazolam is respiratory depression. People with an ABI often have compromised respiratory function [19], some with lower target oxygen saturation ranges, so midazolam must be titrated slowly, and the patients monitored carefully.

This evaluation aimed to review the IV sedation service within the dental department for patients with a profound brain injury at the RHN.

The objectives of this retrospective service evaluation include;

1. To assess the effectiveness of sedation (Ellis sedation scoring and successful completion of exam/treatment) [18]
2. To determine the level of sedation-related complications within this group

## Methods

This study was a retrospective service evaluation of intravenous sedation with midazolam in the dental department at the RHN over seven years. Patients who were treated under IV sedation with midazolam were identified using the department sedation spreadsheet, and data were collected from the clinical records of each patient episode. Information collected included:

- Age
- Gender,
- ASA score,
- Primary cause of brain injury,
- Presence of a tracheostomy,
- Reason for sedation,
- Capacity to consent,
- Justification for sedation
- Baseline oxygen saturation,
- Site of cannulation,
- Midazolam dose,
- Ellis sedation score,
- Treatment performed,
- Sedation-related complications.

The data collected includes patients with Huntington’s disease, an inherited condition that causes damage to the brain. Some data on dental treatment undertaken were missing for some patients; if so, they were excluded from the descriptive analysis of that data.

IBM SPSS 29.0.1.0 was used to complete simple descriptive analysis. Means/standard differentiations are presented for normally distributed continuous data, and medians/interquartile ranges for non-normal data.

## Results

Between 2017 and 2023, 303 patients underwent dental IV sedation at the RHN, of which 122 (40.3%) were female and 181 (59.7%) were male. The patients’ ages ranged between 18 and 83, with a mean of 51 and a standard deviation of 11.8 years. Two hundred seventy-nine patients (92.1%) were assessed as lacking the capacity to consent to dental treatment under sedation at the time of treatment (Table 1).

**Table 1:** Characteristics of the patient population.

	Number of patients, *N* = 303 (%)		
Gender			
Male	181 (59.7)		
Female	122 (40.3)		
Age			
**18**–**19**	1 (0.3)		
20–29	1 (0.3)		
30–39	18 (5.9)		
40–49	108 (35.6)		
50–59	110 (36.3)		
60–69	46 (15.2)		
70–79	13 (4.3)		
80–83	6 (2.0)		
ASA grade			
**1**	0 (0)		
**2**	23 (7.6)		
**3**	279 (92.1)		
**4**	1(0.3)		
Baseline Oxygen Saturation	Mean	Range	Standard deviation
	96%	86–100%	2.5%
Capacity to consent for care			
Yes	24(7.9)		
No	279 (92.1)		
Primary Cause of Brain Injury			
Stroke	112 (37)		
TBI	94 (31)		
Non-TBI	60 (19.8)		
Huntington’s disease	29 (9.6)		
Other	8 (2.6)		
Tracheostomy in situ	40 (13.2)		
Indication for sedation			
Anxiety	20 (2.6)		
Challenging behaviour	186 (61.3)		
Limited mouth opening	62 (20.4)		
Movement	27 (8.9)		
Other	8(2.6)		

Medically, most patients, 92.1% (*n* = 279), were graded as being ASA 3 (Table 1), 14.2% (*n* = 43) had a tracheostomy in situ, and 85.8% (*n* = 260) were fed via an enteral tube. Baseline oxygen saturations ranged from 86–100%, with a median of 96%. The most frequent cause of ABI was stroke (*n* = 112), followed by traumatic brain injuries (*n* = 94), non-traumatic brain injuries (*n* = 60) and Huntington’s disease (*n* = 29). Although stroke is classified as a non-TBI, due to the high prevalence, it was recorded separately (Table 2).

**Table 2:** Ellis grading – a scale used to assess patient cooperation and behaviour during sedation [18].

Ellis grade	Descriptions
I	No interference with treatment; total cooperation and no restlessness
2	Small amount of uninvited movement; still total cooperation and no restlessness
3	More uninvited movement; small degree of restlessness and anxiety. Patient less cooperative but still able to perform all dental procedures
4	Considerable degree of limb movement; perhaps also unhelpful head movements; cooperation poor; patient quite restless and anxious; able to perform only basic dentistry
5	Restlessness, anxiety and limb movements severe; impossible to perform any dentistry

### Sedation details

**Fig. 1:**
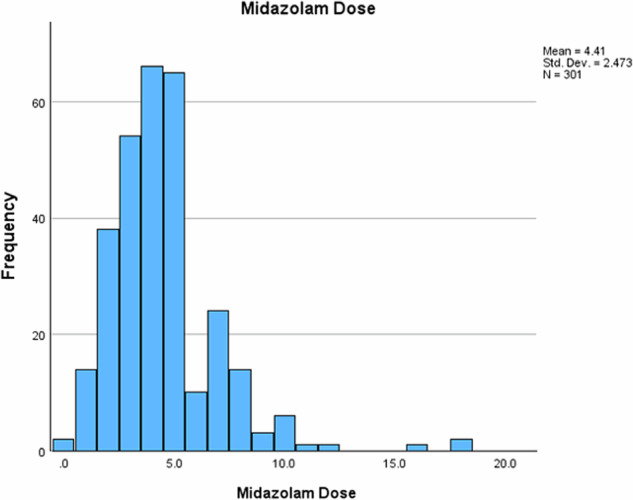
Dose (mg) of midazolam administered.

Cannulation was predominantly performed on the dorsum of the hands (76.5%), with 23.5% in the feet (Table 3). Four patients (1.3%) required 10 mg intranasal midazolam as a premedication via a mucosal atomising device before cannulation. The median IV midazolam dose administered was 4 mg, ranging from zero (where the patient had intranasal midazolam and did not require additional IV midazolam) to 18 mg (Fig. 1).

**Table 3:** Details of sedation.

	Number of patients, *n* (%)		
Cannulation site			
Dorsum of hand	*n* = 232 (76.5)		
Dorsum of foot	*n* = 71 (23.5)		
Premedication needed			
Intranasal midazolam	4 (1.3)		
IV midazolam dose	Median	Range	Interquartile range
	4 mg	0–18 mg	2 mg
Ellis grade			
**1**	55 (18.2)		
**2**	121 (39.9)		
**3**	101 (33.3)		
**4**	24 (7.9)		
**5**	2 (0.7)

As shown in Table 3, 276 (91%) of treatments were graded as Ellis 1, 2, or 3, meaning that the intended treatment in this proportion of the patients was completed. 24 (7.9%) were Ellis 4, where only an examination was possible. Dental treatment was abandoned (Ellis 5) in two (0.7%) cases.

### Dental treatment

Only 208 (68.6%) of patients had data on dental treatment received. Patients missing these data were excluded from the descriptive analysis below. The most frequent dental treatment carried out was a clinical examination (53.4%), followed by dental extractions (42.3%), scaling (27.9%), restorations (23.6%) and radiographs (15.4%). The median number of extractions was two, ranging between zero and five. The median number of restorations was ranging between zero and four. Other treatments performed during sedation included blood tests, botulinum toxin injections to the saliva glands, alginate impressions, jaw relocation, removal of orthodontic appliance, and multidisciplinary care (MDT), including enteral tube changes, ENT or tone assessment (12.5%). All patients who required MDT care primarily required dental assessment or treatment, and the MDT care was carried out opportunistically in the patient’s best interest (Table 4).

**Table 4:** Dental treatment undertaken.

Type of dental treatment	Number of sedation episodes, (*N* = 208)
Dental examination	111 (53.4)
Dental radiographs	32 (15.4)
Scaling	58 (27.9)
Restorations	49 (23.6)
Extractions	88 (42.3%)
Multi-disciplinary care	26 (12.5)

### Sedation related complications

Twenty-four (7.9%) patients experienced minor complications during sedation (Table 5). The most common was a drop in oxygen saturation (*n* = 13), requiring supplemental oxygen during sedation and recovery.

**Table 5:** Sedation relation complications among patients.

Complication	*n* (%)
None	279 (92.4%)
Desaturation requiring supplemental oxygen	13 (4.3%)
Uncontrolled movements	3 (1.0%)
Limited mouth opening not improves with sedation	3 (1.0%)
Large various in pulse rates	2 (0.6%)
Patient pulled out cannula	1 (0.3%)
Prolonged recovery	1 (0.3%)
No reading on pulse oximeter sensor	1 (0.3%)
Disinhibition	1 (0.3%)

No patients required flumazenil to reverse the effect of midazolam either electively or to manage a complication.

## Discussion

IV sedation with midazolam is a valuable technique when providing comprehensive dental examinations and treatment in patients with profound brain injury. When sedating patients with profound ABI compared to patients without a disability, several adjustments need to be made to ensure patient safety. When planning sedation at the RHN, the responsible dentist will always communicate with the patient’s lead medical practitioner to check for any concerns about the proposed treatment. Digital access to all medical records, including clinical observations, is available and provides in-depth patient knowledge, further aiding the assessment of the patient’s suitability for sedation. On the morning of the appointment, the dentist confirms with the nursing team that the patient is medically stable and fit for treatment. If there are any concerns, for example, a raised temperature or recent episodes of oxygen desaturation, the treatment is postponed until they are more stable. When sedating patients with a tracheostomy, an airways-trained medical nurse will be present during the sedation appointment. Airway suction via the tracheostomy is often undertaken before treatment for patients with excessive chest secretions to clear the airways, which will help with breathing and oxygen saturation. A specific mask that fits over the tracheostomy is available to deliver supplemental oxygen if required. The RHN, despite its name, is not a hospital but a specialised centre with a medical presence onsite, including a clinical responsive team, should urgent assistance be needed.

Sedation was carried out following the guidance of the Intercollegiate Advisory Committee on Sedation in Dentistry (IACSD) [17]. Cannulation is mainly undertaken in the hand or foot for this cohort rather than the antecubital fossa due to frequent spasticity and contraction of the arms. Due to the medical complexity of this patient cohort, with 92% graded as ASA3, midazolam was generally titrated as 1 mg over 2 min with an additional 0.5 mg every 4 min. Most patients were administered less than 4 mg of midazolam, lower than in comparable midazolam dental sedation studies [16], due to the need to exercise caution when sedating patients with multimorbidity compared to healthy individuals [20–22]. Sedation appointments tend to be an hour or less. Patients will continue to be monitored as per their usual care back on their ward.

Sedation-related complication rates were low (7.9%), with none of the patients requiring flumazenil to reverse the effects of midazolam. The incidence of oxygen desaturation requiring supplemental oxygen was 4.3%, which is lower than in other dental sedation evaluations [20–22]. Low complication rates are likely due to the slow titration of midazolam, careful sedation planning, and the experience of the dental sedation team. In addition, all patients were sedated in the morning to enable them to be reviewed in the afternoon.

A few studies have suggested that patients may experience transient re-emergence of their stroke  neurological deficits when given midazolam [23, 24]. However, this was not found in any of the 112 cases in this evaluation. Stroke incidence in the UK is projected to increase by 60% between 2015 and 2035, so dental teams will increasingly encounter more stroke survivors requiring dental treatment in primary and secondary care [25].

The Wylie definition of conscious sedation [26] states that verbal contact is maintained with the patient throughout the sedation period. However, many patients with an ABI are not able to maintain verbal contact due to limited or no verbal communication due to their disability. It, therefore, may be difficult for these patients to elicit the level of consciousness and determine when the patient has reached the end point of sedation. A recognised endpoint for dental sedation within the RHN tends to be when the patients accept treatment; this may be when their muscles relax, and they can open their mouths, there is a reduction in anxiety or when involuntary movements reduce. Electroencephalogram (EEG) readings via bispectral index (BIS) monitoring may be an adjunct to clinical monitoring to help determine the sedation endpoint and when patients are fit for discharge. A study by Keddie et al. [27], looked at BIS monitoring for patients with ABI and learning disabilities and found that patients with an ABI took longer for their BIS values to return to baseline compared to patients with a learning disability. Hence, it is important to consider that this cohort of patients often takes longer to recover when deciding when to discharge them from the department.

Challenging behaviour was the most cited indication for sedation, followed by limited mouth opening. A dental examination was the most common procedure undertaken under sedation, reflecting the difficulties in completing comprehensive dental assessments in this specific group of patients. Within this service evaluation, 91% of sedation episodes were graded as Ellis 1, 2 or 3, which indicates that IV sedation is an effective mode of treatment delivery. The Ellis scale was used in this study, as opposed to other sedation scoring methods, which assess the levels of drowsiness and responsiveness to command. Eliciting these responses is often not possible in patients with ABI, especially those with a DOC.

Patients with ABI often have significant dysphagia with unprotected airway reflexes; midazolam has muscle relaxant properties that can further reduce airway patency. To reduce these risks, patients are treated in their wheelchairs and not placed supine, water use during treatment is minimised, good chairside suction is available, and gauze is often placed posteriorly to reduce the risk of aspiration of teeth or debris [28].

Due to the profound nature of their brain injury, a best-interest discussion was undertaken for the majority of patients (92%). Capacity for patients with ABI can fluctuate, and some may regain the capacity to consent as they recover from their injury, so capacity assessments must be reviewed on the day of treatment.

The dental service is unusual because it is commissioned as a primary care contract with no set unit of dental activity (UDA) target. The flexibility of this commissioning model and onsite service provides a patient-centred and cost-effective service. Onsite sedation is less costly than general anaesthesia/anaesthetist-led sedation within a secondary care setting due to lower overhead and staffing costs [29, 30]. It has the additional benefit of enabling multidisciplinary care to be undertaken at the RHN in the patient’s best interest. In some cases, this reduces the need for patients to be referred to a hospital, such as having an enteral tube changed.

All clinicians are consultants in special care dentistry and work with a qualified sedation nurse at the RHN. All of them are experienced in assessing and providing dental care under IV sedation for patients with profound ABIs. The team can discuss cases together for a second opinion, and in some more complex cases, a separate clinician will provide the sedation and dentistry.

Patients post-sedation need to rest and are unable to participate in any of their other therapies for the remainder of the day, which can impact their rehabilitation programme. Remimazolam, an ultra-short-acting benzodiazepine licenced to be used in the United Kingdom since 2021, may offer a safe and effective alternative to midazolam in this patient cohort [31].

The limitations of this evaluation include not collecting data on how many patients desaturated but were managed with patient stimulation and airway manoeuvres. The time it took for the patient to recover after the last increment of midazolam was not recorded. Data regarding dental treatments received were incomplete, with a large number excluded (208/303 episodes included).

## Conclusion

Dentist-led IV sedation with midazolam enables safe and effective dental care to be undertaken for patients with a profound ABI. It is imperative to reflect that these are complex patients with medical issues who are treated in an environment with experienced staff.

People with disabilities face barriers to accessing dental care, and secondary waiting times can be long. Patient-centred sedation services within primary and secondary care must be led by clinicians experienced in treating these patient groups. Service evaluations are important to add to the evidence for safe and effective care.

## Data Availability

The authors confirm that the data supporting the findings of this study are available in the article; further information can be requested from the corresponding author.
